# Can atlas spina bifida-occulta be a cause of cervicogenic headaches?

**DOI:** 10.1186/s40064-015-1395-7

**Published:** 2015-10-13

**Authors:** Amégninou Mawuko Yao Adigo, Lama Kegdigoma Agoda-Kousséma, Ignéza Komi Agbotsou, Kokou Adambounou, Kpalma Duga Bakpatina-Batako, Oni Djagnikpo, Komlanvi Victor Adjénou

**Affiliations:** Radiology Department of Campus Teaching Hospital of Lome, PO Box: 4308, Lome, Togo; Radiology Department of Sylvanus Olympio Teaching Hospital of Lome, Lome, Togo; Neurology Department of Campus Teaching Hospital of Lome, Lome, Togo

**Keywords:** Spina-bifida occulta, Atlas, Cervicogenic headaches, Africa

## Abstract

Cervicogenic headaches are a nosologic entity recently recognized. In our common practice, we have noticed a relative frequency of the atlas spina-bifida occulta during the brain CT scan realized for headaches without cranio-encephalic causes or any other anomaly of the upper cervical region. The aim of this study was to determine a possible connection between cervicogenic headaches (CEH) and atlas spina-bifida occulta. A 2 years prospective and descriptive study in 20 black patients having an atlas spina-bifida occulta diagnosed with a brain CT scan. The mean age of the patients was 43.17 ± 18.35 years (extremes: 24 and 72 years). A light female predominance was noticed (sex-ratio = 1.5). The frequency of symptomatic spina-bifida was 1.72 % (17 cases). The mean age at onset was 31.84 years. The pain was sub-occipital in 14 cases, occipital in 8 cases, bilateral in 12 cases and unilateral in 5 cases. The mean duration of the attacks was 72 ± 24 h and the pain intensity was moderate (16 cases); mean and range were 3.6 and 3–6. The frequency of attacks varied between 1 per 7 months (n = 2) and 2 per week (n = 1) in those with non-daily headache. One attack per 5–7 weeks was the most commonly occurring attack frequency. The pain was reproduced by the pressure of the occipital region or upper cervical in 15 cases. The mean number of criteria was five and there was a strong positive correlation between criteria and CEH (*χ*^2^ = 45.57; V = 0.62). The associated signs were photophobia and nausea in one case each. Indomethacin, Ergotamine and/or Sumatriptan were without any antalgic effect in 16 cases. Pain ceased after an anesthetic blockade of C2 (16 cases). The results show that atlas spina-bifida occulta is not involved in CEH pure form genesis. On a small sample, the atlas spina-bifida seems to be a cause of CEH associated with headache and disorders of the neck.

## Background

Cervicogenic headaches (CEH) are a nosologic entity recently recognized (Sjastaad et al. 1983). They were rare and their diagnostic calls upon anamnestic, clinic and radiologic criteria (Antonaci et al. 2006; Bogduk and Govind 2009). They have numerous causes and comprise the cervico-occipital hinge malformations (Olesen and Steiner 2004). Spina-bifida occulta is a defect of closure of the posterior arc of a vertebra without an individualized paravertebral mass.

In our common practice, we have noticed a relative frequency of the atlas spina-bifida occulta during the brain CT scan realized for headaches without cranio-encephalic causes or any other anomaly of the upper cervical region. But, to our knowledge, no study has established the connection between atlas spina-bifida occulta and headaches. This has motivated us to initiate this study which objective was to determine a possible imputability of the headaches to atlas spina-bifida occulta.

## Patients and methods

### Study design and instruments

It was a 2 years transversal prospective monocentric study from June 2012 to 2014 in black patients. Patients of all age and both sex, referred to the Radiology Department for a brain CT scan, having headaches or not and for who an atlas spina-bifida occulta was observed, were included in our study. All the patients had gone through a rapid malaria diagnostic test which was negative in all the cases. We used a General Electric scanner device (bright speed 16 barrettes). Helical acquisitions were made with and without contrast product. The analysis was made in parenchymatous and bone windows one after the other by two radiologists. There was no discordance and the two radiologists agreed on the normality of the brain CT scan and the upper cervical region except the presence of atlas spina-bifida occulta in all the cases.

We raised as starting hypothesis: can atlas spina-bifida occulta be a cause of CEH?

**Fig. 1:**
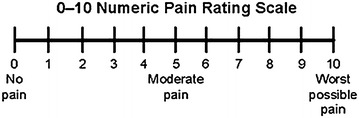
Pain’s intensity evaluation. Graduated ruler permitting an evaluation of the pain by the VAS (Dixit et al. 2013)

During the study, we called the patients who presented a spina-bifida to precise the characteristics of the pain according to CEH criteria (Antonaci and Sjaastad 2011; Sjaastad 2008; Sjaastad and Bakketeig 2008; Fredriksen et al. 2015) without anesthetic blockades. The external digital pressure is exerted with the thumb, at a 90° angle with the skin. Pain intensity was appreciated with the visual analog scale (VAS) and related by Fig. 1 (Dixit et al. 2013). The correspondence between VAS level and pain’s intensity was as follow: from 0 to 3 (low pain); from 3 to 5 (moderated pain); from 5 to 7 (intense pain) et >7 (extremely intense pain).

### Statistical analyses

Qualitative data were treated with Microsoft Word 2007 and with Microsoft Excel 2007. Statistical analyses were performed using R Core Team (2013). The results were tested by Fisher exact test, *χ*^2^ test and Cramer V test. Every difference inferior to 0.05 was considered as significant.

## Results

### Patients’ trends

**Fig. 2:**
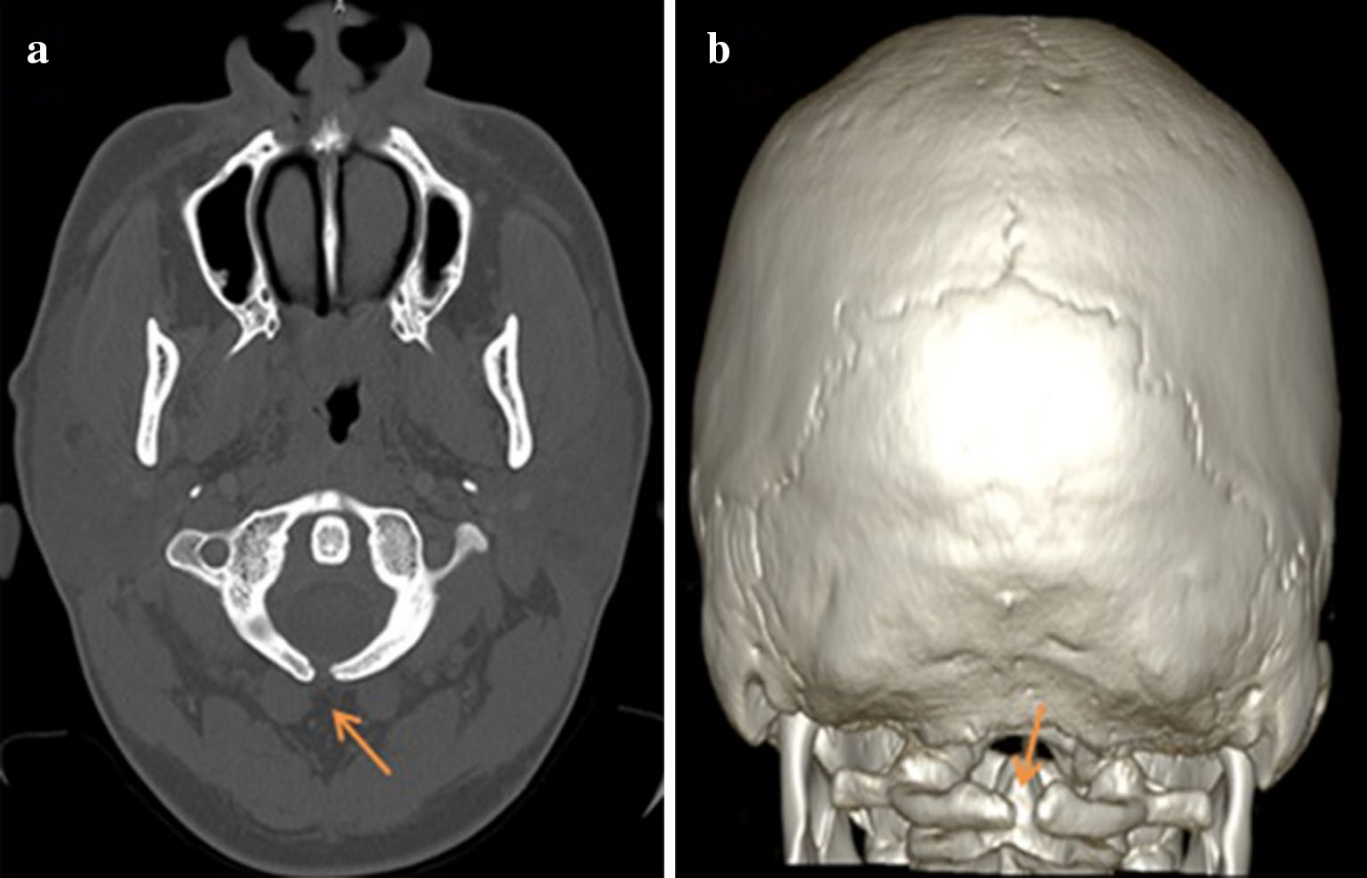
Atlas spina-bifida. Bone windows CT axial cut (**a**) with reconstruction VR (**b**) of an atlas spina-bifida occulta in a patient suffering from headache

Nine hundred and ninety (990) patients were referred in the Radiology Department during the study period. Twenty patients have presented an atlas spina-bifida occulta, 17 were symptomatic, meaning 1.72 % of the brain CT scan realized. Figure 2a, b illustrate the scan aspect of an atlas spina-bifida occulta.

The mean age was 43.17 ± 18.35 years with extremes from 24 to 72 years. A light female predominance came out from our study; the sex-ratio was 1.5. We found a notion of cervical trauma in 11 cases. Primary headache in personal or familial record was found only in 4 cases over 17.

### Pain characteristics

The pain characteristics according to CEH criteria are summarized in Table 1. The difference according to the sex, the various parameters of the pain were statistically significant (p < 0.05). The mean number of criteria was 5.29. There was a strong positive correlation between criteria and CEH (*χ*^2^ = 45.57; V = 0.62).

**Table 1:** The pain characteristics according to cervicogenic headaches criteria

	Male (n)	Female (n)	Total (N)
I: Unilateral head pain, without side shift	3	2	5
II: Provocation, unphysiological neck positions	6	8	14
III: Provocation, externally; neck/occipital area	6	9	15
IV: Range of motion, neck; deficit*	5	8	13
V: Shoulder pain, diffuse	2	3	5
VI: Arm pain, diffuse	1	3	4
VII: Pain, starting posteriorly—ending up anteriorly	7	10	17

* Those with 15° rotation deficit

**Fig. 3:**
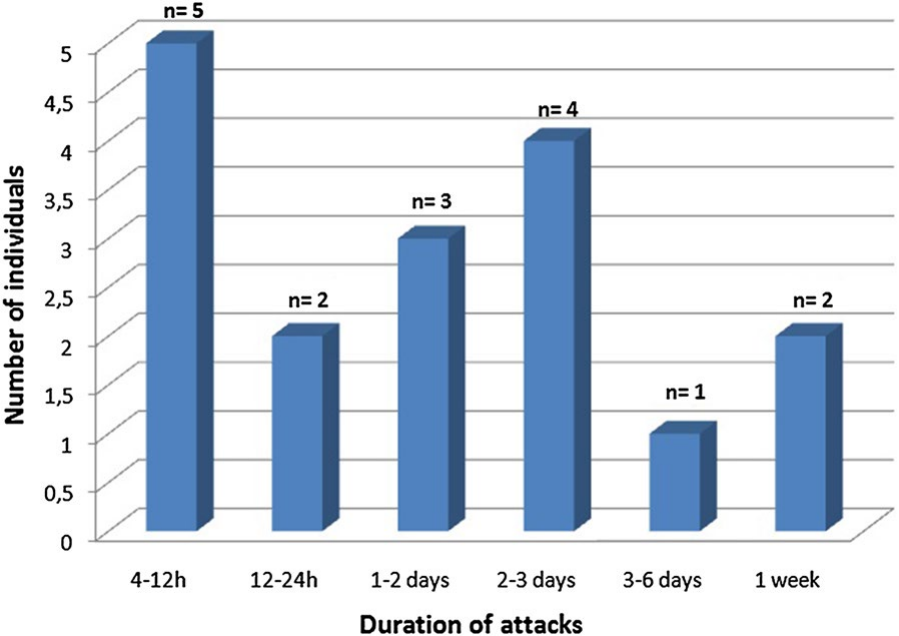
Distribution of the individuals according to the duration of the attacks

The pain was: non-throbbing (16 cases), fluctuating (13 cases) and continuous (4 cases). It was sub-occipital (nape) in 14 cases over 17, occipital in 8 cases, unilateral in 5 cases and bilateral in 12 cases. The mean age (and range) at onset was 31.84 ± 7.35 years (23–47 years). The duration of the attacks is summarized by Fig. 3 (mean = 72 ± 24 h). The pain intensity was moderate (16 cases) or intense (1 case); mean (and range) was 3.6 (3–6). The frequency of attacks varied between 1 per 7 months (n = 2) and 2 per week (n = 1) in those with non-daily headache. One attack per 5–7 weeks was the most commonly occurring attack frequency.

The pressure of the occipital or upper cervical region reproduced the pain in 15 cases over 17 (Table 2). The trigging factors were: inclination of the nape (11 cases) and ante-flexion (14 cases). Cough (10 cases), sneezing (8 cases) and effort (10 cases) were the aggravating factors. The associated signs were photophobia and nausea in one case each. There was no phonophobia, vomiting or periorbital edema.

**Table 2:** Hypersensitive areas of the neck

	Male (n)	Female (n)	Total (N)
Groove behind mastoid process	3	4	7
GON/MON	2	4	6
Transverse processes, C4/C5	2	2	4
Tendon insertions, along bony ridge: protuberantia occipitalis externa, mastoid process	4	5	9
Upper part sternocleidomastoid musclea	4	7	11

Indomethacin, Ergotamine and/or Sumatriptan were without any antalgic effect in 16 cases. Pain regressed after an anesthetic blockades of C2 (16 cases) and spontaneously for one case.

## Discussion

### Prevalence and patients’ trends

The prevalence of CEH was 1.72 % of the brain scanner performed during the study period. This result is lightly inferior to the one (2.2 %) found by Sjaastad and Bakketeig (2008). The difference between both values could be explained by the fact that Sjaastad and Bakketeig (2008) had studied various etiologies of CEH whereas we got focused on the relation between CEH and atlas spina-bifida occulta.

The elements in favor are first of all age and sex. The symptoms started at the middle-age of 31.84 ± 7.35 years. This result corroborates Sjaastad and Bakketeig (2008); Haldeman and Dagenais (2001) studies who have found a middle-age of 33–43 years. The female predominance observed (sex-ratio: 1.5) was evocative of the diagnostic. In the Vågå study (Sjaastad and Bakketeig 2008), there seemed to be a certain male preponderance (female/male: 0.71). In a hospital-based series, however, a female preponderance (2.0–7.2) has been observed (Sjastaad et al. 1983; Sjaastad and Fredriksen 2002). This marked variation has its explanation—a relatively mild CEH form, not leading to consultations, seems to prevail in the population at large. Those consulting physicians are mainly female. In the Vågå study, males, generally, and to a high degree those with CEH, tended to come for an appointment in the final phase. If the study had been interrupted at an earlier stage than at 88.6 %, there would have been a female preponderance also in the Vågå study (Sjaastad and Bakketeig 2008). So, Sjaastad and Bakketeig (2008) thought that the passage in the criteria describing the female sex as a characteristic trait should be deleted.

### Pain characteristics

#### Arguments in favour of the cervicogenic origin

Localization of initial pain was occipital (8/17 cases) and sub-occipital (14/17 cases) with a postero-anterior irradiation in all cases. In fact, the postero-anterior irradiation of the pain could be an important criterion in the diagnostic of CEH according to Fredriksen et al. (2015), International Headache Society (IHS) (2013).

In the majority of the cases, the trigging factors as the inclination of the nape (11/17 cases), ante-flexion (14/17 cases) and the pressure of the occipital or upper cervical region (15/17 cases) were observed. They could allow eliminating in a certain extent a tension headache as an origin of those pains, according to IHS criteria (2013). Digital pressure (i.e., 3–4 kg) directly applied against certain neck structures seems to discriminate fairly well between patients and healthy individuals (Sjaastad and Bakketeig 2008; Sjaastad et al. 2003). A clearly positive test on the symptomatic side is a relatively strong signal for CEH. In the absence of a positive test, at this stage of development, it is hard to establish a CEH diagnosis.

Some elements of our study could allow eliminating migraine attack and tension headache (International Headache Society (IHS) 2013; Antonaci et al. 2006). Those elements were the absence of primary headache in personal or family record, nausea, vomiting and phono/photophobia in the majority of the cases, and the tonality and quality of the headaches (Antonaci et al. 2006; Haldeman and Dagenais 2001). To all this, we can add the absence of pain remission with Indomethacin, Ergotamin and/or Sumatriptan. Chronic paroxysmal hemicrania is easily distinguished from CEH by the following: its absolute response to moderate indomethacin dosages, its relatively short duration, its excessively severe attacks, and its marked autonomic signs (e.g., ipsilateral lacrimation and conjunctival injection) (Sjaastad and Dale 1976).

The mean duration of the attacks was 72 ± 24 h and the pain intensity mean (and range) was 3.6 (3–6). These results corroborate the one found by Sjaastad and Bakketeig (2008) who found a duration superior to 72 h in 61 % of the cases. The mean intensity (and range) of the pain was equal to 3.8 (and 3–5) in Vågå study (Sjaastad and Bakketeig 2008). This mean intensity is lower and bigger than what is observed in migraine without aura (4.2) and tension headaches (3.1); respectively (Sjaastad and Bakketeig 2008). Pain was chronic in all our patients such as in the ‘‘Core’’ group of Sjaastad and Bakketeig (2008).

The mean number of criteria was five and there was a strong positive correlation between criteria and CEH (*χ*^2^ = 45.57; V = 0.62). This mean number of criteria was inferior to Sjaastad and Bakketeig (2008) who found six (6). According to Fredriksen et al. (2015), the five items of IHS (three criteria and two comments) can permit the diagnosis of CEH. Among those five items was the unilaterality of the pain which was the first criterion. This strong correlation coefficient of Cramer shows that there exists a cervicogenic factor in headache genesis in our patients.

The anesthetic blockades of C2 aims diagnostic and therapeutic. It could be a major criterion in the diagnostic of the CEH (Sjastaad et al. 1983; Sjaastad and Bakketeig 2008; Fredriksen et al. 2015; Haldeman and Dagenais 2001; International Headache Society (IHS) 2013; Antonaci et al. 2006). In our study, the quasi-totality of the patients (16/17 cases) had a pain remission after anesthetic blockades of C2. However, the confirmation by anesthetic blockade of C2 or of the greater occipital nerve does not seem specific and are not obligatory in routine work. In fact, Caputi and Firetto (1997) report a significant improvement of 85 % of 23 patients suffering from migraine by anesthetic blockades of the greater occipital nerve.

#### Arguments in disfavor of the cervicogenic origin

Regarding all these arguments evocating a cervicogenic origin of secondary headaches to an atlas spina-bifida occulta, the first unfavorable element was the bilaterality of the pain in the majority of the cases (12/17 cases). In fact, many authors sustain the unilaterality of the pain in the CEH (Sjaastad and Bakketeig 2008; Fredriksen et al. 2015; International Headache Society (IHS) 2013; Antonaci et al. 2006; Caputi and Firetto 1997; Antonaci et al. 2001). Therefore, the frequent bilaterality of the headaches cannot be attributed to a cervicogenic origin and this despite the aggravating circumstances and the positivity of the anesthetic blockade of C2. In the Vågå study, cases presenting with <6 criteria and ≥4 criteria were also grouped together; varying criteria combinations were considered as acceptable evidence for CEH, but unilaterality would still be a demand (Sjaastad and Bakketeig 2008). The great majority of bilaterality found in our study, despite the strong correlation between the criteria and CEH, suggest the existence of another associated factor.

The second argument in disfavor is the small number of diffuse pain of the shoulder and the arm, mean respectively in 4 and 5 cases. Those values are clearly inferior to those observed by Sjaastad and Bakketeig (2008) who found 100 % of the cases for each localization (shoulder and arm).

#### Synthesis of the results

These results on a small sample do not seem to involve the atlas spina-bifida occulta in the genesis of CEH pure form. So, the spina-bifida is, probably, a source of ≪CEH associated with headache and disorders of the neck≫. But could we formally eliminate headaches associated to a cervical rachis disorder pure form? Is the sample sufficient? Perhaps it would be necessary to lead a multicentric study allowing recruiting a greater number of patients, and why not of different races, to confirm the results of this study.

## Conclusion

We thought it would be simple to say that atlas spina-bifida was a source of CEH. We have not found an indisputable connection between spina-bifida occulta of atlas and CEH pure form. Any patient who could have given us this hope was contradicted with the two following. On a small sample, the atlas spina-bifida seems to be a cause of CEH associated with headache and disorders of the neck. A multicentric study on a greater number of patients would allow, maybe, to reinforce the starting hypothesis or eventually to confirm a mixed form.

## Abbreviations

CEH: cervicogenic headaches
IHS: International Headache Society
VAS: visual analog scale
